# Extracellular ATP Increases Glucose Metabolism in Skeletal Muscle Cells in a P2 Receptor Dependent Manner but Does Not Contribute to Palmitate-Induced Insulin Resistance

**DOI:** 10.3389/fphys.2020.567378

**Published:** 2020-09-25

**Authors:** Ana Miguel Cruz, Craig Beall

**Affiliations:** Institute of Biomedical and Clinical Science, University of Exeter Medical School, Exeter, United Kingdom

**Keywords:** ATP, skeletal muscle, insulin sensitivity, palmitate, IL-6, MIF, mitochondrial function

## Abstract

Saturated fatty acids such as palmitate contribute to the development of Type 2 Diabetes by reducing insulin sensitivity, increasing inflammation and potentially contributing to anabolic resistance. We hypothesized that palmitate-induced ATP release from skeletal muscle cells may increase inflammatory cytokine production and contribute to insulin/anabolic resistance in an autocrine/paracrine manner. In C2C12 myotubes differentiated at physiological glucose concentrations (5.5 mM), palmitate treatment (16 h) at concentrations greater than 250 μM increased release of ATP and inflammatory cytokines IL-6 and MIF, significantly blunted insulin and amino acid-induced signaling and reduced mitochondrial function. In contrast to our hypothesis, degradation of extracellular ATP using apyrase, did not alter palmitate-induced insulin resistance nor alter release of cytokines. Moreover, treatment with ATPγS (16 h), a non-hydrolysable ATP analog, in the absence of palmitate, did not diminish insulin sensitivity. Acute treatment with ATPγS produced insulin mimetic roles; increased phosphorylation of PKB (aka AKT), S6K1 and ERK and enhanced GLUT4-mediated glucose uptake in the absence of exogenous insulin. The increases in PKB and S6K1 phosphorylation were completely prevented by pre-incubation with broad spectrum purinergic receptor (P2R) blockers PPADs and suramin but not by P2 × 4 or P2 × 7 blockers 5-BDBD or A-438079, respectively. Moreover, ATPγS increased IL-6 yet decreased MIF release, similar to the cytokine profile produced by exercise. Acute and chronic treatment with ATPγS increased glycolytic rate in a manner that was differentially inhibited by PPADs and suramin, suggesting heterogeneous P2R activation in the control of cellular metabolism. In summary, our data suggest that the palmitate-induced increase in ATP does not contribute to insulin/anabolic resistance in a cell autonomous manner.

## Introduction

Lipid overflow, particularly of saturated free fatty acids, contributes to the pathophysiology of Type 2 Diabetes (T2D) by reducing insulin sensitivity and enhancing inflammation and mitochondrial dysfunction (Wu and Ballantyne, 2017; Petersen and Shulman, 2018; Sergi et al., 2019). These impairments are predominant in skeletal muscle, resulting in loss of glucose clearance that accounts for 85–90% of the impairments in glucose homeostasis observed in T2D (Katz et al., 1983; Pendergrass et al., 2007). Lipid-induced muscle insulin resistance (IR) is brought about primarily by impaired insulin signaling due to build-up of lipotoxic species and due to increased levels of circulating pro-inflammatory cytokines. Lipotoxic moieties associated with the development of IR include ceramides (Summers, 2006), long-chain fatty acyl-coenzyme A (Ellis et al., 2000), diacylglycerol (Itani et al., 2002) and acylcarnitines (Aguer et al., 2015), among others [recently reviewed by Bergman and Goodpaster (2020)]. Excess lipid availability also appears to negatively regulate anabolic sensitivity, demonstrated by anabolic resistance in overweight and obese individuals (Guillet et al., 2009; Murton et al., 2015; Beals et al., 2016); in humans following a continuous intralipid infusion (Stephens et al., 2015), in diet-induced obese rodents (Anderson et al., 2008; Masgrau et al., 2012) and in high-fat fed men in a model of disuse-induced IR (Wall et al., 2020).

A proportion of skeletal muscle inflammation occurs due to immune cell infiltration of muscle adipose depots (Khan et al., 2015). Independently from this, treatment with saturated fatty acids, particularly palmitate, as the major dietary fatty acid in blood, enhances the release of cytokines such as interleukin 6 (IL-6) and activates the IKK/NF-κB signaling cascade (Jove et al., 2005), which is associated with the development of skeletal muscle IR. IL-6 has been extensively characterized for its paradoxical roles in inflammation. Acute and transient exercise-induced increases in IL-6 contribute to the regulation of glucose homeostasis, for example, by stimulating glucose production (Fischer, 2006; Pedersen and Febbraio, 2008; Wolsk et al., 2010; Eckardt et al., 2014), glucagon secretion (Barnes et al., 2014) and altering adipose tissue function (Brandt et al., 2012). In contrast, chronically elevated circulating IL-6 features in pro-inflammatory conditions and is associated with muscle wasting and IR (Kern et al., 2001; Pradhan et al., 2001). Other cytokines and myokines, such as macrophage migration inhibitory factor (MIF), are elevated in inflammatory conditions such as obesity (Dandona et al., 2004) and T2D (Yabunaka et al., 2000) and have modulatory roles in glucose homeostasis (Atsumi et al., 2007; Miyatake et al., 2014). It remains important to establish the autocrine and paracrine roles of these myokines in skeletal muscle IR and inflammation.

Palmitate can also enhance muscle ATP release in a pannexin hemi-channel-mediated manner (Pillon et al., 2014). Palmitate-induced extracellular ATP (eATP) acts as a chemoattractant for immune cells, suggesting that the nucleotide plays an important role in the immune cell infiltration seen in conditions characterized by chronic low-grade inflammation, such as obesity and T2D (Wu and Ballantyne, 2017). In other tissues, eATP (and degradation products) has been implicated in inflammation and IR, as seen in adipose tissue (Tozzi and Novak, 2017; Wu and Ballantyne, 2017); in blockade of insulin receptor signaling in hepatocytes by adenosine diphosphate (ADP) (Chatterjee and Sparks, 2012) and by impaired glucose clearance and hepatic insulin sensitivity in mice lacking the ectonucleotidase CD39 (Enjyoji et al., 2008).

ATP is released from skeletal muscle in response to electrical stimulation (Buvinic et al., 2009; Bustamante et al., 2014) and by contraction/exercise (Steensberg et al., 2000; Osorio-Fuentealba et al., 2013). eATP acts in an autocrine/paracrine manner by signaling via metabotropic P2Y and ionotropic P2X purinergic receptors (P2Rs) leading to increased intracellular calcium concentration ([Ca^2+^]_i_) (Cseri et al., 2002; Abdul-Ghani et al., 2008; Ito et al., 2018) and transactivation and transinhibition of pathways such as MAPK/ERK and protein kinase C signaling cascades (Ito et al., 2018). eATP also acts as an important vasodilator (Mortensen et al., 2009) and likely contributes to muscle hypertrophy following exercise (Ito et al., 2018). Given the well-characterized pro-inflammatory roles of eATP in other tissues and enhanced ATP release following treatment with saturated fatty acids, we hypothesized that palmitate-induced increases in eATP may contribute to the development of insulin/anabolic resistance and inflammation in an autocrine/paracrine manner in skeletal muscle, in the absence of immune cells.

## Materials and Methods

### Chemicals

Novo Nordisk Actrapid recombinant human insulin was used for cell treatments (Henry Schein, United Kingdom). ATPγS, PPADs, suramin, 5-BDBD, A-438079 were purchased from Tocris (Bristol, United Kingdom). Fluo-4 Direct and 50 x MEM amino acid solution were from Thermo Fisher Scientific (Loughborough, United Kingdom). ATP (adenosine 5’-triphosphate magnesium salt), apyrase, fatty acid-free BSA, palmitic acid and indinavir were purchased from Sigma Aldrich (Poole, United Kingdom).

### Cell Culture

The C2C12 (ATCC CRL-1772, *Mus musculus*) murine myoblast cell line was kindly gifted by colleagues at the University of Dundee. Cell were maintained in growth medium below 70% confluence, incubated at 37°C in humidified 5% CO_2_ incubators. Cells were differentiated at physiologically relevant glucose concentrations (5.5 mM). For differentiation, cells were plated in plating medium and incubated for 48 h (reaching 100% confluence) before medium was changed to differentiation medium. Differentiation medium was changed every day for 6 consecutive days to replenish glucose. Experiments were conducted after 7 days of differentiation. Refer to Supplementary Methods for media composition and seeding densities.

### Immunoblotting

Experiments in 60 mm dishes were terminated by media collection (for ATP and cytokine analysis) and cell lysis for protein extraction. Protein concentrations were determined by the Bradford method and lysates subjected to SDS-PAGE and electrotransferred to nitrocellulose membranes. Total and phosphorylated proteins and loading controls were immunoblotted and semi-quantified by infrared fluorescence using the Licor Odyssey scanner. See Supplementary Methods for lysis buffer and antibody details.

#### Cytotoxicity Assessment

Cell viability was assessed in C2C12 myotubes using extracellular lactate dehydrogenase (LDH) activity measured using a commercially available kit and according to manufacturer’s instructions (LDH assay kit, Abcam, United Kingdom). Briefly, C2C12 myoblasts were differentiated as above for 6 days and treated with palmitate (0–750 μM) or BSA controls for 16 h before LDH activity was measured. Medium collected after overnight treatment was centrifuged (600 × *g* for 10 min) and supernatants incubated with WST and lactate dehydrogenase. LDH oxidizes lactate (producing NADH) which reacts with WST to generate a yellow color which was detected by absorbance (OD_450__nm_) using Pherastar FS plate reader.

### ATP Quantification

The extracellular concentration of ATP was measured using a commercially available assay kit and used according to manufacturer’s instructions (ATPLite, PerkinElmer) and as previously described (Vlachaki Walker et al., 2017). See Supplementary Methods for details.

### Assessment of Cellular Metabolism

Mitochondrial and glycolysis stress tests were performed according to manufacturer’s instructions with minor modifications (Agilent, United Kingdom). Briefly, for mitochondrial stress tests, cells were treated for 16 h with palmitate or BSA controls and treated acutely with with oligomycin (2 μM), FCCP (1 μM) and rotenone/antimycin A (1:1 ratio, final concentration 1 μM). For glycolysis stress tests, cells were treated with ATPγS (up to 16 h) and treated acutely with glucose (10 mM); oligomycin (2 μM) and 2-deoxyglucose (2-DG, 50 mM). Refer to Supplementary Methods for details.

### Cytokine Quantification

The levels of extracellular cytokines present in conditioned media from treated cells were quantified using enzyme linked immunosorbent assays (ELISAs). MIF and IL-6 were measured using mouse Duo-set ELISA kits (Bio-Techne, Abingdon, United Kingdom), performed according to manufacturer’s instructions.

### Measurement of Intracellular Calcium

Changes in intracellular calcium were assessed using the fluorescent calcium indicator Fluo-4 Direct as previously described (Ito et al., 2018), with minor modifications for the C2C12 cells. Briefly, C2C12 myotubes were differentiated in clear, flat bottomed 96 well plates. After 7 days, cells were incubated with Fluo-4 Direct-containing phenol red free medium for 60 min and relative changes in fluorescence measured using a Pherastar FS plate reader. See Supplementary Methods for details.

### Measurement of Glucose Uptake

Briefly, C2C12 myotubes were differentiated in 96-well plates for 7 days (as above). For palmitate experiments, cells were treated for 16 h with palmitate (500 μM) or BSA control on day 6. On day 7, cells were serum starved for 2 h and glucose starved for 1–2 h before incubation with treatments and subsequent incubation with 2-DG (100 μM, 15 min). 2-DG uptake into C2C12 myotubes was assessed using the Glucose Uptake-Glo assay (Promega, Southampton, United Kingdom). See Supplementary Methods for details.

### Statistical Analyses

In Western blotting experiments, a One−sample *t*−test was used to determine significant changes in phosphorylation or total protein expression, relative to control (normalized to 1). For multiple group comparisons, a one−way or two-way analysis of variance with *post hoc* Bonferroni were used. Statistical tests were performed using GRAPHPAD PRISM software (Prism 7; GraphPad Software, La Jolla, CA, United States). Results are expressed as mean ± standard error. *P* values < 0.05 were taken to indicate statistical significance.

## Results

### Palmitate Treatment Diminishes Insulin Sensitivity and Increases ATP Release

**FIGURE 1:**
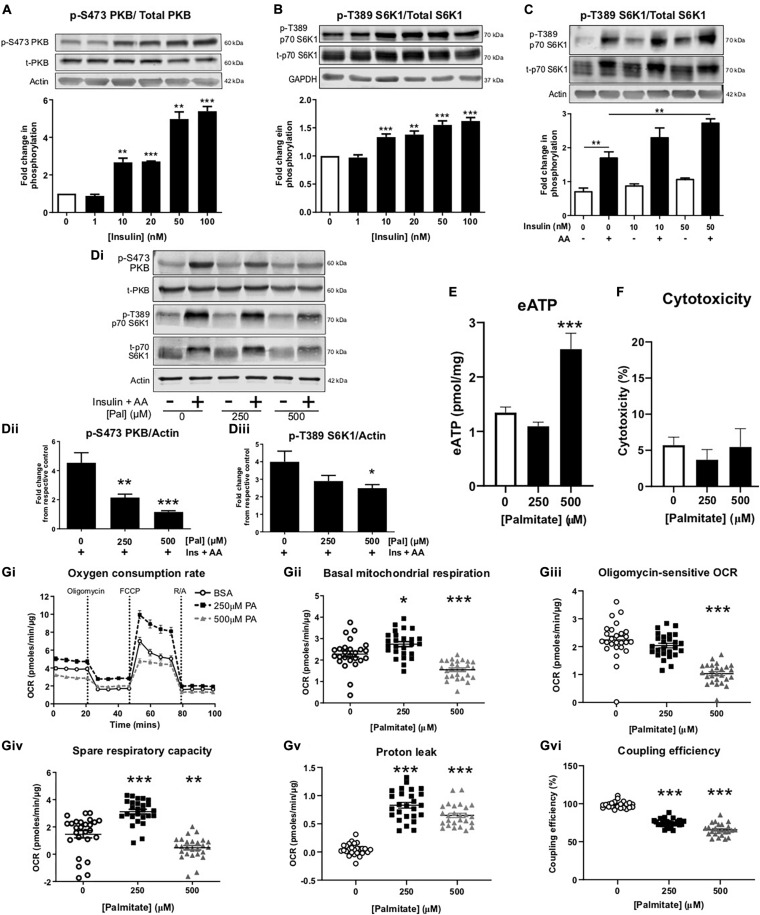
Palmitate induces insulin resistance, enhances extracellular ATP and impairs mitochondrial function in C2C12 myotubes. Representative immunoblots for PKB (pS473) normalized to PKB **(A)** and p70-S6K1 (pT389) normalized to p70-S6K1 **(B)** with densitometric analysis below for cells treated with insulin (0–100 nM) for 60 min (*n* = 4–7). Actin and GAPDH used as loading controls. **(C)** Representative immunoblot for p70-S6K1 (pT389) normalized to p70-S6K1 for cells treated for 60 min with amino acids (AA, 3.34 mM) or combined with insulin (0–50 nM) (*n* = 3). **(Di)** Representative immunoblots for PKB (pS473) and p70-S6K1 (pT389) normalized to actin with densitometric analysis in **(Dii,iii)**, respectively, represented as fold change from respective control, for cells treated with palmitate (0–500 μM; 16 h) and insulin (20 nM) and amino acids (AA, 3.34 mM) for 60 min (*n* = 5–6). **(E)** Extracellular ATP measured by luminescence after palmitate treatment (0–500 μM; 16 h; *n* = 7). **(F)** Cytotoxicity assessed by extracellular lactate dehydrogenase in palmitate (0–500 μM; 16 h) treated myotubes (*n* = 3). **(Gi)** Representative extracellular flux assay demonstrating oxygen consumption rate (OCR; pmoles/min/μg) at baseline and after acute injection of oligomycin (2 μM), FCCP (1 μM) and rotenone/antimycin A (R/A; 1:1 ratio; 1 μM) at the indicated stages (*n* = 26–28). **(Gii)** Mean basal OCR before oligomycin. **(Giii)** Oligomycin-sensitive OCR measured as effect of oligomycin. **(Giv)** Spare respiratory capacity, measured as FCCP effect. **(Gv)** Proton leak measured as R/A effect. **(Gvi)** Coupling efficiency measured as the ratio between oligomycin-sensitive OCR and mitochondrial basal OCR expressed as percentage. *P < 0.05, **P < 0.01, ***P < 0.001 against untreated control.

Differentiation of myoblasts into myotubes at physiologically relevant glucose concentrations yielded myotubes that were sensitive to insulin (10–100 nmol/L), as measured by significant increases in PKB and S6K1 phosphorylation (Figures 1A,B). In addition, treatment with mixed essential amino acids (AA) enhanced S6K1 phosphorylation (Figure 1C). In the absence of palmitate, insulin and AAs significantly increased the levels of phosphorylated PKB and S6K1, however, in the presence of 500 μM palmitate, insulin/AA-induced PKB and S6K1 phosphorylation was significantly attenuated (Figures 1Di–iii). We observed no change in extracellular ATP (eATP) levels with 250 μM palmitate, but at 500 μM palmitate, eATP levels were elevated approximately two-fold (Figure 1E). Treatment with 500 μM palmitate did not alter cell viability (Figure 1F). As expected, palmitate treatment led to significant impairments in mitochondrial function with cells displaying reduced basal oxygen consumption rates (OCR; Figures 1Gi,ii), reduced oligomycin-sensitive OCR (a measure of ATP production; Figures 1Gi,iii), diminished spare respiratory capacity (Figures 1Gi,iv), increased proton leak (Figures 1G,Gv) and reduced coupling efficiency (Figure 1Gvi).

### Degradation of eATP Does Not Alter Palmitate-Induced Insulin Resistance and Cytokine Release

**FIGURE 2:**
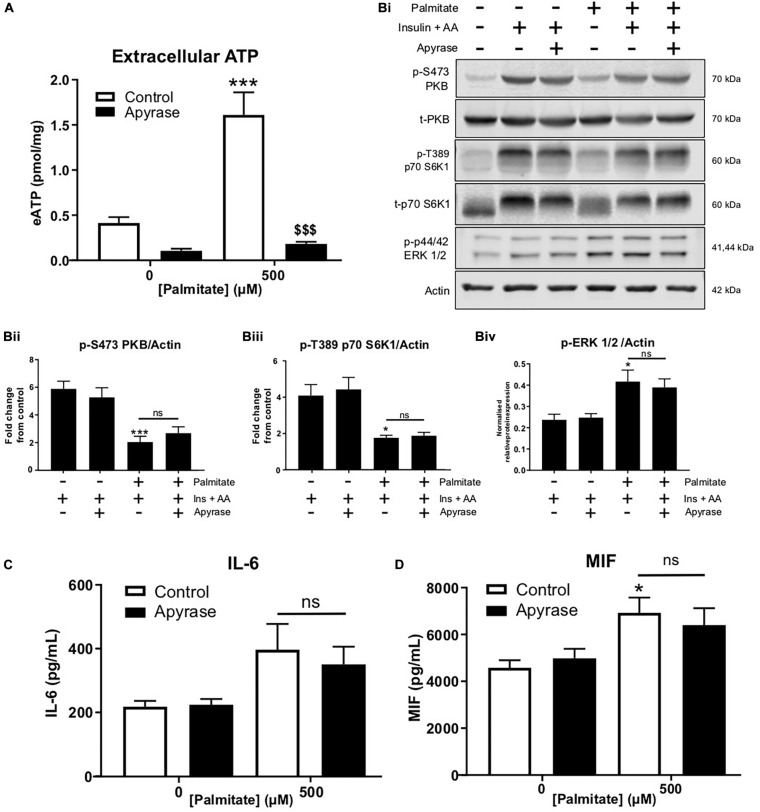
Palmitate-induced insulin and anabolic resistance and cytokine release is not altered by hydrolysis of extracellular ATP with apyrase in C2C12 myotubes. **(A)** Palmitate-induced extracellular ATP (500 μM; 16 h) hydrolysed by treatment with apyrase (0.5 units/mL; 16 h) in cells treated for 15 h with PA + apyrase and 1 h with insulin (20 nM) and amino acids (3.34 mM) (*n* = 6). **(Bi)** Representative immunoblots for PKB (pS473), total PKB, p70-S6K1 (pT389), p70-S6K1, ERK 1/2 (pThr202/Tyr204) and actin (*n* = 6) for cells treated as in A. Densitometric analysis for PKB (pS473) normalized to PKB **(Bii)** p70-S6K1 (pT389) normalized to p70-S6K1 **(Biii)** and ERK 1/2 (pThr202/Tyr204) normalized to actin **(Biv)**. **(C,D)** Hydrolysis of extracellular ATP with apyrase (0.5 units/mL; 16 h) did not alter the palmitate (500 μM; 16 h)-induced increase in release of cytokines IL-6 (C; *n* = 6) or MIF (D; *n* = 5). *P < 0.05, ***P < 0.001 for palmitate against control and ns = not significant for the effect of apyrase on palmitate response. ^[*dollar*][*dollar*][*dollar*]^*P* < 0.001 against palmitate treated group.

In separate studies, we confirmed that palmitate, at pro-inflammatory concentrations, enhanced eATP and that apyrase degraded palmitate-induced eATP by 90% (Figure 2A). Palmitate treatment significantly blunted insulin and AA-mediated phosphorylation of PKB and S6K1, however, apyrase treatment did not alter blunting of insulin/AA-mediated signaling (Figures 2Bi–iv). Palmitate significantly increased ERK1/2 phosphorylation and this was not altered by apyrase, suggesting that additional mechanisms contribute to the palmitate-induced phosphorylation of ERK1/2 (Figures 2Bi, iv). We noted a modest increase in IL-6 and a significant increase in macrophage migration inhibitory factor (MIF) induced by palmitate but this was not significantly modified by apyrase (Figures 2C,D). Taken together these data indicate that eATP does not contribute to palmitate-induced insulin resistance nor does it significantly contribute to palmitate-induced cytokine release from skeletal muscle.

### ATPγS Treatment in the Absence of Palmitate Does Not Alter Insulin Sensitivity but Does Alter Cytokine Secretion

**FIGURE 3:**
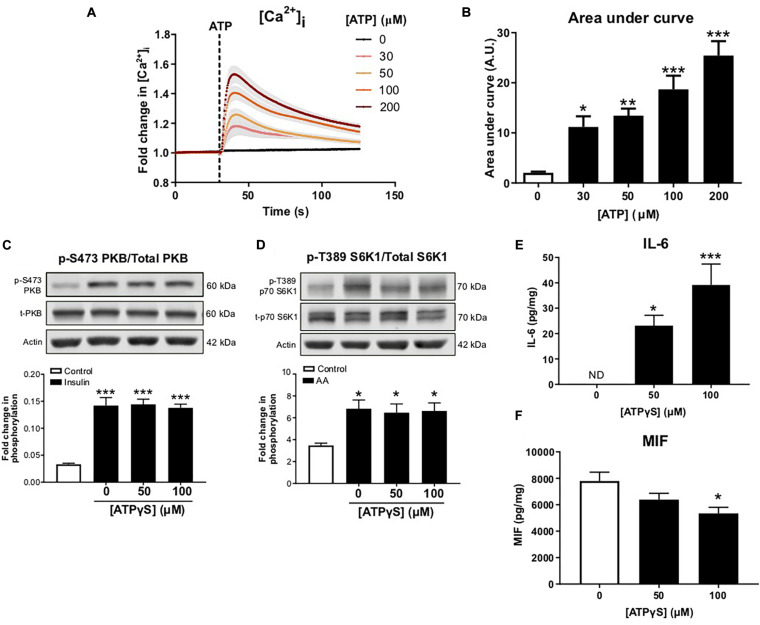
Treatment with exogenous ATP increases intracellular calcium and alters cytokine release without impairing anabolic sensitivity in C2C12 myotubes. **(A)** Cells loaded with Fluo 4 direct for 60 min. Concentration-dependent increase in intracellular calcium assessed by change in fluorescence (RFU) with ATP (0–200 μM; 30 s) with area under the curve in **(B)** (*n* = 6). **(C)** Representative immunoblot and densitometric analysis for PKB (pS473) normalized to PKB following 16 h treatment with 50–100 μM ATPγS and 1 h treatment with insulin (20 nM) or control (*n* = 6). **(D)** Representative immunoblot and densitometric analysis for p70-S6K1 (pT389) normalized to p70-S6K1 following 16 h ATPγS treatment and 1 h treatment with mixed essential amino acids (AA, 3.34 mM) or control (*n* = 6). **(E,F)** Exogenous ATPγS (50–100 μM; 16 h) enhanced IL-6 release (E; *n* = 6) and suppressed MIF release (F; *n* = 6). *P < 0.05, **P < 0.01, ***P < 0.001 against vehicle control.

To confirm ATP-induced signaling in C2C12 myotubes, intracellular calcium responses were monitored following ATP treatment. ATP caused a concentration-dependent increase in intracellular calcium, indicating P2R-mediated signaling (Figures 3A,B). To determine whether enhanced P2R-mediated signaling could alter insulin sensitivity, in the absence of palmitate, myotubes were incubated with a non-maximal concentration of ATPγS (based on changes to intracellular calcium levels), a non-hydrolysable ATP analog, for 16 h prior to acute insulin treatment. As expected, acute insulin treatment increased PKB and S6K1 phosphorylation but this was not modified by ATPγS pre-treatment (Figures 3C,D). Interestingly, when measuring release of IL-6 and MIF from C2C12 cells treated with ATPγS, we observed a significant concentration-dependent increase in IL-6 release (Figure 3E). In contrast to the palmitate response, however, we observed a significant reduction in MIF release following ATPγS treatment (Figure 3F).

### Acute ATPγS Treatment Increases PKB and S6K1 Phosphorylation in a P2R-Dependent Manner and Enhances GLUT4-Mediated Glucose Uptake in Healthy but Not Palmitate-Challenged Cells

**FIGURE 4:**
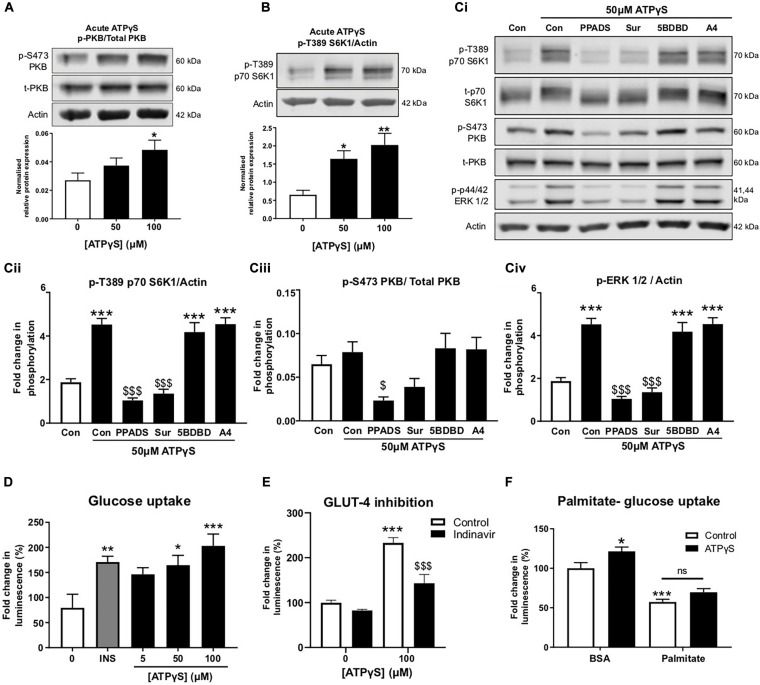
ATPγS has insulin-mimetic roles in C2C12 myotubes activating PI3K/PKB and mTOR pathway signaling proteins in a P2-receptor-dependent manner and enhancing GLUT4-mediated glucose uptake. **(A,B)** Representative immunoblots for PKB (pS473) normalized to PKB (A; *n* = 5) and p70-S6K1 (pT389) normalized to p70-S6K1 (B; *n* = 6) with densitometric analysis below for cells treated with ATPγS (50–100 μM; 15 min). **(Ci)** Representative immunoblots for PKB (pS473), PKB, p70-S6K1 (pT389), p70-S6K1, ERK 1/2 (pT202/TY204) and actin for cells treated with ATPγS (50 μM; 15 min) with and without broad spectrum P2 receptor antagonists PPADs (100 μM) and suramin (100 μM), P2X4R antagonist 5-BDBD (5 μM) and P2X7R antagonist A438079 (100 μM) (45 min prior to spiked ATPγS; *n* = 6). Densitometric analysis for p70-S6K1 (pT389) normalized to p70-S6K1 in **(Cii)**; PKB (pS473) normalized to PKB in **(Ciii)** and ERK 1/2 (pT202/TY204) normalized to actin in **(Civ)**. **(D)** Glucose uptake (2-DG; 100 μM; 15 min) assessed by luminescence following treatment with ATPγS (0–100 μM; 15 min pre-treatment) or insulin (200 nM; 15 min pre-treatment) (*n* = 9). **(E)** Glucose uptake (2-DG; 100 μM; 15 min) following treatment with ATPγS (100 μM; 15 min) in the presence or absence of GLUT4 inhibitor indinavir (50 μM) (*n* = 5). **(F)** Glucose uptake (2-DG; 100 μM; 15 min) following treatment with palmitate (500 μM; 16 h) and ATPγS (100 μM; 15 min) (*n* = 17). *P < 0.05; **P < 0.01; ***P < 0.001 against untreated control, $$$*P* < 0.001 against ATPγS control.

To examine ATPγS signaling in more detail, C2C12 myotubes were treated acutely with ATPγS and PKB and S6K1 phosphorylation was examined. Correlating with recent studies (Osorio-Fuentealba et al., 2013; Ito et al., 2018), we observed significant increases in phosphorylation of both PKB and S6K1 following ATPγS treatment (Figures 4A,B). To determine which P2Rs may be responsible, myotubes were pre-incubated with broad-spectrum P2R blockers PPADs and suramin, in addition to P2 × 4 and P2 × 7 antagonists, 5-BDBD and A-438079. Interestingly, PPADs significantly blocked ATPγS-induced increases in PKB, S6K1 and ERK1/2 phosphorylation while suramin significantly attenuated the latter two and modestly but not significantly attenuated PKB phosphorylation. Moreover, basal phosphorylation was also reduced, indicating tonic receptor activation (Figures 4Ci–iv). Acute treatment with ATPγS enhanced glucose uptake in a concentration dependent manner, independently of insulin (Figure 4D). In addition, pre-treatment with GLUT4 inhibitor indinavir attenuated ATPγS-mediated glucose uptake by 40% (Figure 4E). To assess whether ATPγS-mediated glucose uptake was sustained in insulin resistant myotubes, we assessed glucose uptake following palmitate treatment and demonstrated that palmitate significantly attenuated basal and ATPγS-stimulated glucose uptake (Figure 4F).

### ATPγS Increases Glycolytic Metabolism by a P2R-Dependent Mechanism

**FIGURE 5:**
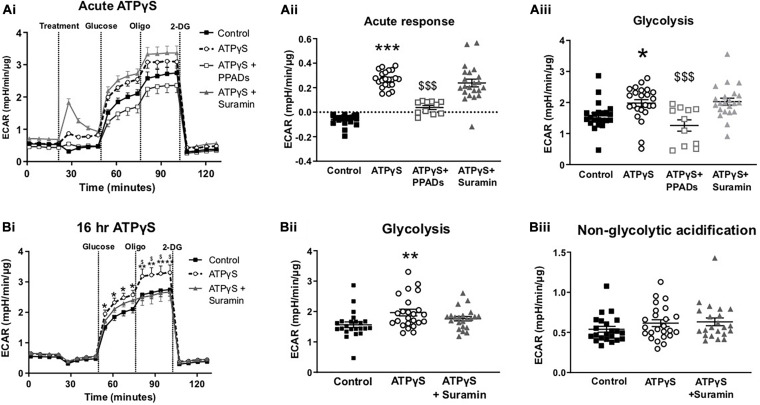
Treatment with ATPγS enhances extracellular acidification rate in C2C12 myotubes in a P2-receptor-dependent and insulin-independent manner. **(Ai)** Representative extracellular flux assay demonstrating changes to glycolytic rate as estimated by extracellular acidification rate (ECAR) in response treatment with ATPγS (100 pM; treatment) with or without suramin (100 pM), PPADs (100 pM) or vehicle; followed by acute injection of glucose (5.5 mM), oligomycin (2 pM) and 2DG (50 mM) at the indicated points (*n* = 22–24). **(Aii)** Acute response, ECAR prior to glucose **(Aiii)** Glycolysis measured as the change in ECAR following glucose injection. **(Bi)** Representative extracellular flux assay for cells treated with ATPγS (100 pM; 16 h) with or without suramin (100 pM) or vehicle and acute injection of glucose (5.5 mM), oligomycin (2 pM) and 2-DG (50 mM) at the indicated points (*n* = 22–24). **(Bii)** Glycolysis measured as the change in ECAR following glucose injection. **(Biii)** Non-glycolytic acidification demonstrating mean ECAR prior to glucose injection. Data represented as mean ± SEM. *P < 0.05; **P < 0.01; ***P < 0.001 against control, $*P* < 0.05, $$$*P* < 0.001 against ATPγS.

To examine whether the enhancement in glucose uptake led to altered intracellular glucose metabolism, we performed a glycolysis stress test in the presence and absence of ATPγS and P2R antagonists. Acute injection of ATPγS induced a modest but statistically significant increase in the extracellular acidification rate (ECAR), in the absence of glucose (Figures 5Ai,ii). This response was abolished by PPADs but not by suramin, indicating this acute response is unlikely to be mediated by purely the addition of ATPγS. In the presence of suramin, ATPγS generated a large increase in ECAR (Figures 5Ai,ii). This was not accompanied by increased oxygen consumption rate (OCR; data not shown) suggesting this is not mediated by CO_2_ production from the mitochondria. On addition of glucose, ATPγS treatment augmented the ECAR response, suggesting enhanced glycolysis. Again, this was blocked by PPADs, but not by suramin (Figures 5Ai,iii). We next examined the effect of chronic (16 h) ATPγS treatment on glycolytic function. Correspondingly, ATPγS significantly enhanced glycolysis. In this paradigm, pre-incubation with suramin prevented the enhanced ECAR response to glucose injection by ATPγS, producing ECAR levels similar to control (Figures 5Bi,ii). Non-glycolytic acidification (before injection of glucose) was not altered by ATPγS pre-treatment (Figure 5Biii).

## Discussion

Skeletal muscle insulin resistance contributes substantially to the pathophysiology of T2D. Defining the pathways of insulin resistance and uncovering potential mechanisms to improve insulin sensitivity are key therapeutic areas requiring further investigation. It has previously been reported that palmitate induces release of ATP in skeletal muscle, which can recruit immune cells (Pillon et al., 2014). In liver, saturated fatty acids also increase hemi-channel expression to increase ATP release which can stimulate apoptosis (Xiao et al., 2012). Moreover, the action of ATP at P2Rs is a potent modulator of pro-inflammatory cytokine production and release in a number of non-immune cells including keratinocytes (Kawano et al., 2015), endothelial cells (Seiffert et al., 2006) and astrocytes (Liu et al., 2000). Therefore, given the role of ATP in mediating pro-inflammatory signaling, we postulated that ATP release from skeletal muscle, in an autocrine/paracrine manner and independently of immune cell recruitment, may contribute to production of pro-inflammatory cytokines and reduced insulin sensitivity in palmitate challenged myotubes. Here we utilized the C2C12 myoblast cell line, differentiated into myotubes, to observed ATP release and exogenous ATP signaling in a cell autonomous manner. We demonstrate that palmitate induces ATP release from skeletal muscle myotubes independently of cell death, assessed by measurement of extracellular lactate dehydrogenase, agreeing with previous reports demonstrating palmitate-induced ATP release in a regulated manner (Pillon et al., 2014). In our study, palmitate-induced ATP release was accompanied by reduced insulin/AA sensitivity, diminished mitochondrial function and increased release of cytokines IL-6 and MIF. Contrary to our hypothesis, degradation of eATP using apyrase did not modify palmitate-induced insulin resistance nor cytokine release. In contrast, enhanced eATP signaling (in the absence of palmitate) produced insulin mimetic actions; increasing PKB, S6K1 and ERK1/2 phosphorylation. Recently, Ito and colleagues observed eATP-mediated increases in S6K1 and protein synthesis in C2C12 myotubes, mediated by the P2Y2 receptor (Ito et al., 2018). Our findings are in line with these observations and suggest that eATP has anabolic actions in muscle.

We also observed increased GLUT4-mediated glucose uptake into C2C12 myotubes following ATPγS treatment, in line with previous reports (Osorio-Fuentealba et al., 2013). Exercise significantly increases ATP demand/turnover in muscle (Gaitanos et al., 1993) and palmitate-induced mitochondrial dysfunction leads to reduced ATP production (Nisr and Affourtit, 2016) and, interestingly, ATP release occurs in both situations (Osorio-Fuentealba et al., 2013; Pillon et al., 2014). Contraction and exercise-induced ATP release contributes to glucose uptake and cytokine release in skeletal muscle (Osorio-Fuentealba et al., 2013; Bustamante et al., 2014). If sensitivity to ATP-induced glucose uptake is sustained in insulin resistant states, this may contribute to exercised-induced anabolism and provide a novel therapeutic strategy to maintain glucose homeostasis in T2D. We tested the acute response to ATPγS following palmitate treatment, mimicking contraction-induced ATP release in insulin resistant conditions, and demonstrated attenuated ATP-simulated uptake in palmitate-challenged cells, suggesting a level of purinergic resistance. Resistance to ATP-mediated glucose uptake has been demonstrated at lower ATP concentrations in muscle fibers from high-fat fed mice, but not at higher ATP concentrations, where response is sustained {Osorio-Fuentealba et al., 2013 #5679}. Additional experimentation is required to investigate purinergic sensitivity in metabolically impaired skeletal muscle. However, others have demonstrated diminished P2R responses to ATP in insulin resistant conditions, such as dampened P2Y2R-mediated vasodilation in T2D (Thaning et al., 2010), indicating that purinergic resistance could also occur at the level of glucose uptake and metabolism.

Our novel observations that ATPγS enhances skeletal muscle cell glycolytic function suggests an important receptor-mediated process to increase ATP generation as a positive feedback mechanism. Similar increases in cellular metabolism have been reported previously in endothelial cells, where exogenous ATP increased ECAR and upregulated key glycolytic enzymes including hexokinase, glucose transporter 1 expression and phosphofructokinase B3 (Lapel et al., 2017). This effect of eATP on metabolism is likely tissue specific as eATP has been reported to decrease cellular oxygen consumption in kidney cells, in a manner sensitive to suramin (Silva and Garvin, 2009). It is also worth noting that PPADs and suramin differentially blocked the effect of ATPγS on metabolism in this study, indicating that both P2X and P2YRs may contribute to glucose uptake and/or metabolism. Interestingly, blockade of G-protein signaling only partially attenuated ATP-induced glucose uptake (Osorio-Fuentealba et al., 2013), suggesting a component of glucose transport could be P2XR mediated. However, it is important to note that purinergic receptor and ATPase expression can change during differentiation (Martinello et al., 2011) and varies across species (Abdelmoez et al., 2020), therefore determining the receptor(s) in human skeletal muscle going forward will be important.

In our study, ATPγS also increased IL-6 and decreased MIF release from myotubes. In general, transient and short-term muscle-derived IL-6, such as seen during exercise, promotes hypertrophy, myogenesis and regeneration (Fischer, 2006). IL-6 can also enhance skeletal muscle glucose uptake and fat oxidation via AMP-activated protein kinase dependent and independent pathways (Carey et al., 2006; Wolsk et al., 2010; Ikeda et al., 2016). Conversely, long-term elevation of IL-6, which is evident in inflammatory conditions, is associated with muscle wasting and IR (Kern et al., 2001; Pradhan et al., 2001; Rui et al., 2002). Therefore it is plausible that persistent fatty acid-induced ATP release drives chronic IL-6 signaling to contribute to IR in an autocrine/paracrine fashion, in addition to the recruitment of immune cells (Pillon et al., 2014). Therefore the time course and persistence of the ATP release likely contributes to the differential response to acute (exercise-induced) versus chronic (palmitate-induced) ATP release. For example, a sustained elevation in eATP may be required to recruit and polarize immune cells toward a pro-inflammatory phenotype (Desai and Leitinger, 2014; Pillon et al., 2014). Furthermore, the physiological (increased ATP demand during contraction) and pathological context (impaired ATP production during excess lipid) may alter the outcome of eATP signaling, which requires further investigation.

Interestingly, ATPγS, in the absence of palmitate, decreased MIF release. MIF is a pro-inflammatory cytokine that is packaged into readily releasable pools (Bacher et al., 1997). The circulating levels of MIF are elevated in T2D (Yabunaka et al., 2000), Type 1 Diabetes (Bojunga et al., 2003; Ismail et al., 2016) and a number of other autoimmune diseases (Ayoub et al., 2008; Vincent et al., 2019). In addition, deletion of MIF in pancreatic beta cells protects them against palmitate-induced apoptosis (Saksida et al., 2012). In adipose tissue, MIF can inhibit insulin-stimulated glucose uptake (Atsumi et al., 2007) and in skeletal muscle, MIF can reduce insulin and AICAR-mediated glucose transport (Miyatake et al., 2014). Therefore, the significant decrease in MIF release following ATPγS treatment suggests a possible beneficial effect of ATPγS.

Although not yet explored, it is also plausible that the benefits of neuromuscular electrical stimulation (NMES) in preventing disuse-induced atrophy during immobilization (Dirks et al., 2014) are partly attributed to the autocrine anabolic roles of eATP. This contraction-induced ATP release to the muscle interstitium (Mortensen et al., 2009) is mirrored by a transient increase in plasma ATP which increases and returns to basal levels within 30 min of recovery (Zarêbska et al., 2018). Utilizing ATP as a signal in this manner has a number of advantages specifically in muscle. For example, muscle contraction causes plasma membrane damage (McNeil and Khakee, 1992), leading to release of intracellular contents (including ATP); differentiated muscle cells express high levels of ATPases (Martinello et al., 2011) that rapidly degrade eATP allowing swift termination of the signal; and eATP can contribute to enhanced blood flow and oxygenation into muscle (Rosenmeier et al., 2008; Mortensen et al., 2009), which is essential during exercise. Whether this can be exploited as a therapy remains to be determined. Distinguishing physiological versus pharmacological actions of ATP will be important going forward, particularly during different states such as exercise or nutrient excess. However, in humans, oral ATP supplementation has been reported to increase muscle blood flow (Jäger et al., 2014) and size following resistance training (Wilson et al., 2013). It can also promote hypotension after exercise in hypertensive women (de Freitas et al., 2018). The reasons for the benefits of oral ATP remain unknown given that the bioavailability of oral ATP is very low (Arts et al., 2012) and that direct infusion of ATP into the femoral vein failed to increase interstitial ATP concentrations (Mortensen et al., 2009), which suggests that oral ATP itself is unlikely to be useful for improving glucose control. Whether a small molecular P2R agonist, delivered systemically, could replicate the benefits of eATP reported here will be important to determine. A better understanding of the purinergic regulation of glucose uptake, metabolism and cytokine release in conditions with compromised insulin and anabolic sensitivity is required to assess therapeutic potential of this system.

In summary, we show that extracellular ATP release does not contribute to reduced insulin sensitivity induced by palmitate in skeletal muscle myotubes but does increase glucose uptake and metabolism by a P2R-dependent mechanism. Importantly, the cytokine profile generated by ATP/ATPγS is similar to the signature generated by moderate and high intensity exercise, which has been shown to increases muscle and plasma ATP levels; increase skeletal muscle glucose uptake (Carey et al., 2006; Ikeda et al., 2016); increase circulating levels of IL-6 (Sprenger et al., 1992) and to decrease circulating MIF levels (Wahl et al., 2014). The potential for the exercise mimetic actions of ATP and P2R modulation in skeletal muscle to treat conditions such as T2D requires further study.

## Data Availability Statement

The raw data supporting the conclusions of this article will be made available by the authors, without undue reservation.

## Author Contributions

AC designed and performed experiments, analyzed data and contributed to interpretation of data and writing of the manuscript. CB conceived the study, contributed to experimental design and wrote the manuscript. Both authors agree to be accountable for the content of the work.

## Conflict of Interest

The authors declare that the research was conducted in the absence of any commercial or financial relationships that could be construed as a potential conflict of interest.
