# Supplementary material for: Extracellular ATP Increases Glucose Metabolism in Skeletal Muscle Cells in a P2 Receptor Dependent Manner but Does Not Contribute to Palmitate-Induced Insulin Resistance
Source: Front Physiol. 2020 Sep 25;11:567378. doi: 10.3389/fphys.2020.567378 (PMC7545032; doi:10.3389/fphys.2020.567378)
Supplement: Supplementary file 1 [file Table_1.docx]

**Supplementary methods**

***Cell culture***

C2C12 myoblast media requirements and seeding densities in Tables 1 and 2, respectively. Cells were maintained sub-confluent (60 – 70 %) in culture in growth medium. For differentiation, cells were plated according to seeding densities and volumes on Table 2, in plating medium, and incubated for 48 hrs at 37 °C, 5% CO_2_. After 48 hrs (100 % confluence) medium was replaced with differentiation medium. Medium was changed every day for 6 consecutive days. Overnight treatments were conducted in serum-deprived amino acid poor medium (Table 1). All experiments were conducted in serum-deprived medium.

| **Medium** | **Components** |
| --- | --- |
| Growth medium | High glucose (25 mM) DMEM supplemented with 10 % FBS, 4 % (v/v) L-glutamine, 2 % penicillin/streptomycin (pen/strep) |
| Plating  medium | Glucose-free DMEM with 4 % L-glutamine supplemented with 5.5 mM glucose, 10 % FBS and 2 % pen/strep |
| Differentiation medium | Glucose-free DMEM with 4 % L-glutamine supplemented with 5.5 mM glucose, 2 % horse serum and 2 % pen/strep |
| Amino acid-deprived medium (EBSS) | Earl’s balanced salt solution 1 x, supplemented with sodium bicarbonate (2.2 g/L) and 0.34 mM AA solution. |
| Seahorse XF medium – mitochondrial stress test | XF DMEM with 5.5 mM glucose, 2.5 mM sodium pyruvate, 2 mM L-glutamine, pH 7.4 |
| Seahorse XF medium – glycolysis stress test | Glucose-free XF DMEM with 2 mM L-glutamine, pH 7.4 |

**Table 1. Media composition for C2C12 myoblast culture and differentiation**

***Table 2. Seeding density and volumes for C2C12 myoblast culture***

| **Culture platform** | **Density per well/dish** | **Plating**  **volume** | **Differentiation volume (mL)** | **Experimental volume (mL)** |
| --- | --- | --- | --- | --- |
| T75 flask | 1 x 10^6^ | 10 mL | - | - |
| 60 mm dish | 2 x 10^5^ | 5 mL | 3 mL | 1.5 mL |
| F-bottom 96 well plate | 5 x 10^3^ | 200 µL | 200 µL | 50-200 µL |
| Seahorse XFe96 microplate | 3 x 10^3^ | 200 µL | 200 µL | 180 µL |

***Immunoblotting***

Lysis buffer used to lyse cells was comprised of: 25 mM Tris HCL pH 7.4, 50 mM sodium fluoride, 100 mM sodium chloride, 1 mM EDTA pH 8, 1 % (v/v) Triton 100x, 10 mM tetrasodium pyrophosphate supplemented with 268.7 mM sucrose, 0.1 % (v/v) 2-mercaptoethanol, 1 mM sodium orthovanadate, 1 mM benzamidine and 1 mM phenylmethylsulfonyl fluoride.

Primary antibodies used were: pS473 PKB (1:1000; #9271); total PKB (1:1000; # 9272); pT389 p70S6K1 (1:1000; #9205); total p70S6K1 (1:000; # 9202); p44/42 MAPK (ERK1/2) (1:1000; #4370) from Cell Signaling Technologies. Total β-Actin (1:10000; #NB600-501) from Bio-techne and GAPDH (1:10000; #G9545) from Sigma Aldrich. Secondary antibodies used were: Alexa Fluor 680 goat anti-mouse IgG (1:10000; #A21057) from Thermo Fisher and Anti-IgG (Rabbit) Goat polyclonal Ab (1:10000; #ROCK611-132-122 from VWR. Bands were quantified using Image Studio Lite (v4) and densitometric analysis carried out by normalising phospho to total bands or to actin/GAPDH as loading controls.

***ATP Quantification***

Extracellular ATP was quantified using ATPLite kit (PerkinElmer) according to manufacturer’s instructions. Briefly, conditioned media from palmitate treated C2C12 myotubes (0-250 µM; 16 hrs) was assayed by addition of 50 µL of mammalian cell lysis solution (MCLS) to 100 µL of sample (in triplicate) in black-walled 96-well plates and gently shaking for 5 minutes. ATP substrate solution (50 µL) was added to the well and plate mixed for 5 minutes before being transferred to a plate reader (Pherastar FC, BMG Labtech, UK) for a 10 minute dark adaptation period. Luminescence was read and ATP concentrations calculated against a standard curve of known ATP concentrations (0-100 pmoles) on each plate. This bioluminescence-based assay uses a firefly luciferase-mediated reaction between ATP and d-luciferin. The emitted light from the reaction below is directly proportional to ATP concentration.

magnesium, luciferase

ATP + D-Luciferin + O_2_  Oxyluciferin + AMP + PPi + CO_2_ + Light

ATP levels were normalised to the total protein content (quantified using the Bradford method) of the cell monolayer from which the medium was collected.

***Glucose uptake***

Glucose uptake was assessed using Glucose Uptake-Glo assay (Promega), according to manufacturer’s instructions. This non-radioactive plate-based assay uses a bioluminescence method to detect 2-deoxyglucose-6-phosphate (2DG6P), which is formed following 2-DG uptake into cells and cannot be metabolised. C2C12 myotubes were differentiated in 96-well plates for 7 days (as above). For palmitate experiments, on day 6, cells were treated with palmitate (500 µM) or fatty acid free BSA (1 % w/v) in serum free 5.5 mM glucose medium for 16 hr prior to glucose uptake assessments on day 7. For other experiments, on day7, cells were incubated for 2 hrs in serum free DMEM (5.5 mM glucose) at 37 °C.

For palmitate experiments, after 14 hr incubation, medium was replaced with serum free, glucose free DMEM supplemented with palmitate (or BSA control) and cells incubated for 2 hrs at 37 °C. For acute ATPγS experiments, after serum free incubation, medium was replaced with serum free, glucose free DMEM and cells incubated for 1 hr at 37 °C. Cells were then pre-treated with treatment of interest (insulin, ATPγS, indinavir or vehicles) and subsequently incubated for 15 minutes with 100 μM 2-DG (dissolved in PBS). Reaction was terminated by acid detergent solution and pH neutralised with high-pH buffer solution. A detection reagent containing glucose-6-phosphate dehydrogenase (G6PDH), NADP+, reductase, luciferase and proluciferin substrate was then added. Luminescence emitted from the luciferin based reaction was detected with 0.3-1 second integration using the PHERAstar FS microplate reader.

***Assessment of cellular metabolism***

For mitochondrial stress tests and glycolytic stress tests (Agilent, UK), C2C12 myoblasts were differentiated (as above) in Seahorse XFe96 microplates at 3 x 10^3^ cells/well. For palmitate treatments, cells were treated on day 6 with palmitate (250 – 500 µM) conjugated to fatty-acid free BSA (0.2 % w/v) or BSA control for 16 hrs in serum free Earl’s balanced salt solution (supplemented with sodium bicarbonate 2.2 g/L and 0.3 mM mixed amino acid solution). Seahorse XFe96 sensor cartridges were hydrated with Seahorse XF calibrant solution and maintained at 37 ^o^C in a non-CO_2_ incubator overnight. On day 7, medium was replaced with low buffered Seahorse XF medium (Table 2) supplemented with treatments (as per 16 hr incubation) and cells incubated for 1 hr in non-CO_2_ incubator, at 37 °C for 1 hr. After incubation, plates were loaded onto Seahorse XFe96 Analyzer and basal oxygen consumption rate (OCR) measured for 4 cycles. After basal measurements, cells were injected with the following every 4 cycles: Oligomycin (final concentration 2 μM), FCCP (final concentration 1 μM) and Rotenone/Antimycin A (1:1 ratio, final concentration 1 μM). Measurement were taken every 6 minutes on a 3 minute mix, 3 minute measure cycle. For glycolytic stress tests cells were treated on day 6 with ATPγS (0-100 µM) +/- suramin or acutely with ATPγS (spiked during assay) and assay performed as above with the following differences: de-gas step was performed in XF medium deprived of glucose and sodium pyruvate and supplemented with broad spectrum purinergic receptor antagonist PPADS (100 µM) or suramin (100 µM) or controls. Extracellular acidification rate (ECAR) was measured for 4 baseline cycles and injection strategy initiated. Cells were injected with ATPγS or control (dH_2_O) followed by a saturating concentration of glucose (10 mM), oligomycin (2 µM) and 2-DG (50 mM), every 4 cycles.

Following completion of all assays, media was removed and cells lysed with sodium hydroxide (100 µL per well of 50 mM NaOH). Protein concentrations were quantified using the Bradford method. OCR and ECAR readings were normalised to total protein concentration in each well.

***Measurement of intracellular calcium***

Changes in intracellular calcium were assessed using the fluorescent calcium indicator Fluo-4 Direct. C2C12 myoblasts were plated in clear, flat bottomed 96 well plates at a seeding density of 5x10^3^ cells/well in plating medium and differentiated (as above) for 7 days. On the day of the experiment, cells were serum starved for up to 2 hours before being incubated with Fluo-4 Direct-containing phenol red free media (PRF, supplemented with 25 mM HEPES and 5.5 mM glucose) for 60 minutes at 37^o^C. Following incubation, fluorescence was captured using a PHERAstar FS plate reader with 485 nm and 520 nm excitation and emission wavelengths, respectively. Measurements were recorded every 0.2 seconds with compounds used to stimulate calcium responses being injected after 30 seconds. Cells were acutely injected with ATP stock solution (10X in PRF medium) or control (dH_2_O in PRF) to achieve final concentrations in well between 0 – 200 µM. Changes in calcium were expressed as relative fluorescent units normalised to a baseline value of 1.
